# Supplementary material for: The causal role of circulating inflammatory markers in osteoporosis: a bidirectional Mendelian randomized study
Source: Front Immunol. 2024 Jul 18;15:1412298. doi: 10.3389/fimmu.2024.1412298 (PMC11291241; doi:10.3389/fimmu.2024.1412298)
Supplement: Supplementary file 1 [file DataSheet_1.zip › supplementary material/CIM-OSTEOPOROSIS /GCST90274848.csv_leave_one_out.pdf]

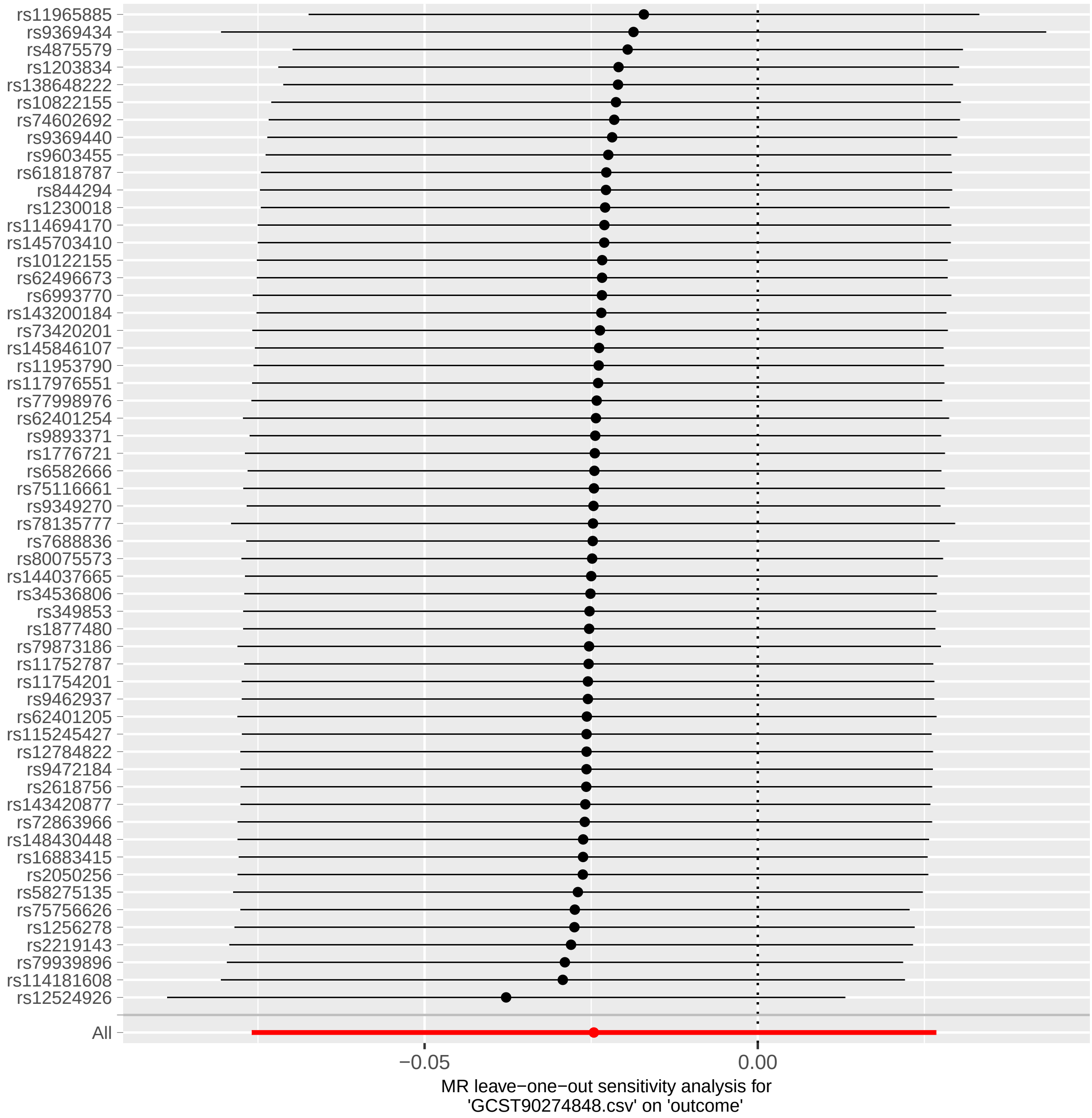
rs11965885
rs9369434
rs4875579
rs1203834
rs138648222
rs10822155
rs74602692
rs9369440
rs9603455
rs61818787
rs844294
rs1230018
rs114694170
rs145703410
rs10122155
rs62496673
rs6993770
rs143200184
rs73420201
rs145846107
rs11953790
rs117976551
rs77998976
rs62401254
rs9893371
rs1776721
rs6582666
rs75116661
rs9349270
rs78135777
rs7688836
rs80075573
rs144037665
rs34536806
rs349853
rs1877480
rs79873186
rs11752787
rs11754201
rs9462937
rs62401205
rs115245427
rs12784822
rs9472184
rs2618756
rs143420877
rs72863966
rs148430448
rs16883415
rs2050256
rs58275135
rs75756626
rs1256278
rs2219143
rs79939896
rs114181608
rs12524926
All
−0.05
0.00
MR leave−one−out sensitivity analysis for
'GCST90274848.csv' on 'outcome'
